# TIP, TORQUE & ROTATIONS: How accurately do digital superimposition software packages quantify tooth movement?

**DOI:** 10.1186/s40510-022-00402-x

**Published:** 2022-03-14

**Authors:** Samar M. Adel, Nikhilesh R. Vaid, Nadia El-Harouni, Hassan Kassem, Abbas R. Zaher

**Affiliations:** 1 Department of Orthodontics, Faculty of Dentistry, Alexandria University, Champollion Street, El Azarita, Alexandria, Egypt; 2 Department of Orthodontics, Saveetha Dental College, Saveetha Insitute of Medical and Technical Sciences, Chennai, India

**Keywords:** Artificial intelligence, Registration, 3D digital models, 3D tooth movement, Digital orthodontics, Aligner therapy, Scanning, Digital setup

## Abstract

**Background:**

To investigate the accuracy of three different 3D digital model registration software for tip, torque and rotation measurements, with reference to a 3D digital virtual setup. Twenty maxillary and mandibular pre-treatment scans of patients undergoing clear aligner therapy were used. Digital setups were generated from pre-treatment scans using a tooth movement software. Both the pretreatment digital scans (T1) and digital setups (T2) were converted to STL files to be exported to the 3 studied software that employed: (1) Semiautomatic best fit registration (S-BF), (2) Interactive surface-based registration (I-SB), and (3) Automatic best fit registration (A-BF) respectively. Changes in tip, torque and rotation were calculated for all the registered pairs.

**Results:**

The change in tooth position was compared between the calculated tooth movement using each of the registration software packages versus the actual generated tooth movement from the digital setups. Continuous data was expressed as mean and standard deviation. Intra Class Correlation Coefficient for agreement between digital simulation and each software was used. Intra and Inter-examiner reliabilities were also assessed using Intra Class Correlation Coefficient. Significance of the obtained results was expressed at *p* ≤ 0.01. Semiautomatic best fit registration software showed excellent agreement (> 0.90) for all tooth movements, except for good agreement for torque (0.808). Interactive surface-based registration software showed moderate agreement for all measurements (0.50 and < 0.75), except for good agreement for rotation (0.783). Automatic best fit registration software demonstrated excellent agreement (> 0.90) for rotation, good agreement for tip (0.890) and moderate agreement for torque (0.740).

**Conclusions:**

Overall, semiautomatic best fit registration software consistently showed excellent agreement in superimpositions compared to other software types. Automatic best fit registration software consistently demonstrated better agreement for mandibular superimpositions, compared to others. Accuracy of digital model superimpositions for tooth movements studied in superimposition studies, can be attributed to the algorithm employed for quantification.

## Background

Digital superimpositions are integral to quantifying tooth movement effects in contemporary orthodontic protocols, where movement simulations are employed for designing orthodontic appliances. Through this appraisal, the clinician can understand capabilities and limitations of appliances and mechanics employed [1–4]. Clear Aligner Therapy (CAT) is one of the most robust applications of digital technology, where tooth movement is programmed to a simulation. When teeth are assigned a target position through virtual planning, tracking and quantifying their movements through treatment becomes integral to therapeutic success [5].

Digital intraoral models derived either from model scans or direct intraoral scans are the first step in obtaining a detailed 3D representation of the dentition, on which planning, measurements and simulations are performed [6–9]. Tooth movement can be studied by registering serial 3D models acquired at different time points where they can be combined in the same spatial coordinate system [10]. Variable techniques and software packages have been used for 3D digital registration of virtual models as well as for tooth movement measurements, so as to quantify treatment between time periods. These software packages differ in the registration methods they offer, in the method of measuring 3D tooth movements, in their costs, in time taken, and in complexity to perform a specific task [11–13].

Most available software packages for model registration use a combination of computer-based Artificial Intelligence (AI) algorithm and operator data-input [14–16]. These AI algorithms can be classified based on the degree of interaction, the transformation domain, and most importantly the type of algorithm employed (Table 1) [17–19] So far, there is no consensus in the literature regarding the techniques to superimpose serial 3D intraoral digital models [3]. Several limitations exist in current literature comparing different registration techniques with regards to the standard reference used [3, 11, 20–24].

**Table 1:** Software packages employed and their mode of operation [16]

Classification criteria	3D Digital Model Registration Software		
	Semiautomatic best fit registration software (S-BF)	Interactive surface- based registration software (I-SB)	Automatic best fit registration software (A-BF)
Degree of interaction	Semi-automatic	Interactive	Automatic
Transformation domain	Global	Local	Both
Method of registration	Surface based (Best fit method)	Landmark based/selected area	Information theory and mathematical algorithm technique-based (Best fit method)
Algorithm used	Iterative Closest Point Algorithm	Non-Iterative Algorithm	Iterative Closest Point Algorithm

Studies have used AI based software packages which register digital models, assess 3D tooth movements, quantify treatment effects and assess appliance efficacy. However, the changes expressed in those studies are dependent on how accurate is the given software employed.

The aim of this study was to evaluate the accuracy of three different AI 3D digital model registration software packages that quantify tip, torque and rotation to a predetermined simulated 3D digital setup. The null hypothesis was that there is no agreement between the predetermined tooth movement generated by the digital setup and the different AI registration software packages.

## Materials and methods

### Study design

This diagnostic accuracy and agreement study followed a modification of the Guidelines for Reporting Reliability and Agreement Studies (GRRAS) where each software package was considered as a rater [25]. IRB approval was obtained from the Faculty of Dentistry, Alexandria University (IRB: 00010556-IORG: 0008839) and informed consents sought from the subjects whose scans were used as a study material. Access to the original scans was limited to the principal investigator. All potentially identifiable patient information was removed from the scans. The minimal sample size was calculated based on previous studies that aimed to evaluate the reliability of newly developed software calculating 3D tooth movement [12, 26]. Based on the results, a sample size of 20 scans was deemed enough to conduct this agreement study [27], with minimum accepted reliability *ρ*_0_ = 0.6 and maximum expected reliability *ρ*_1_ = 0.9, *k* = 3, where k corresponds to the number of tested software packages. The statistical significance alpha was set at 0.01 to account for multiple comparisons and a statistical power, 1-*β* = 0.9. The minimum calculated sample size was 18, increased to 20 to account for defective scans.

### Sample collection

The sample of this study consisted of full arch pretreatment maxillary and mandibular intraoral digital scans of actual adult patients undergoing CAT. All scans were randomly selected from the records of a single orthodontic office in Mumbai, India with more than 15 years of experience with CAT. A random number list of 20 was generated using Microsoft Excel from the total number of scans available in the office archive. The scanner used was a TRIOS 3-D intraoral scanner (3Shape, Copenhagen, Denmark). The scan data was then exported in STL format file extension and the files were imported into the three studied software and analyzed in the Department of Orthodontics, Alexandria University. The study group comprised scans of 20 patients with a Little’s irregularity index that ranged from 4-6 mm. All teeth in both arches were evaluated for 3D angular tooth movements except for third molars. The inclusion criteria for the scans were (1) Adult subjects treated with CAT who received treatment in both arches, (2) Scans had to be complete and of acceptable quality with a full complement of teeth except for the third permanent molars. Scans were excluded if (1) Treatment involved extraction of permanent teeth, (2) Teeth had surface anomalies or if (3) Scans had soft-tissue lesions covering the palate or the mucogingival junction (MGJ) of the mandibular arch.

**Fig. 1:**
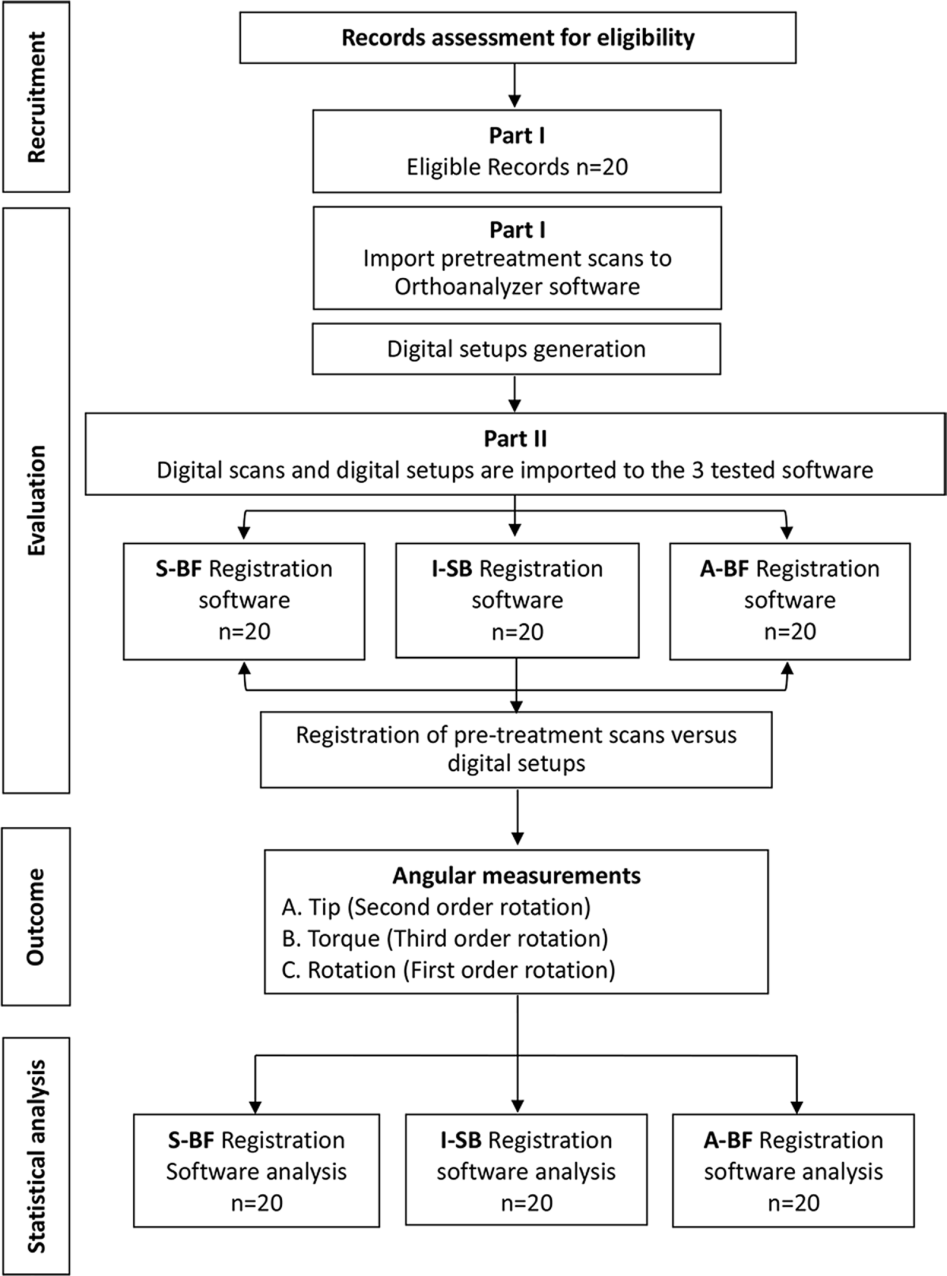
Research Flowchart

All the scans that met the eligibility criteria were given an identification number. All digital scans were de-identified by an independent investigator, and imported into the 3 different tooth measuring software programs for the principal investigator to evaluate Fig. 1.

### Procedure

#### Digital setup

Full arch maxillary and mandibular pretreatment scans (T1) were imported to OrthoAnalyzer software (3Shape Ortho System, Copenhagen, Denmark). Virtual digital setups were done by using virtual segmentation techniques. All tooth movements were visualized and quantified in all directions. Tip, torque and rotation measurements of this Digital Setup (DS) were tabulated for all teeth and used as reference for measuring accuracy of the 3 different software. The DS were exported as STL model files and termed (T2).

T1 and T2 models were  imported as STL files to the tooth measuring software programs, for registration and 3D angular measurements. The three studied software packages were:

1. *Semiautomatic best fit registration software (S-BF)*: Geomagic (Geomagic U.S., Research Triangle Park, NC) using landmark based method followed by regional global surface registration [17].
2. *Interactive surface-based registration software (I-SB)*: OrthoAnalyzer (3Shape Ortho System, Copenhagen, Denmark) using surface 3-point method of registration [18].
3. *Automatic best fit registration software (A-BF)*: eModel 9.0 “Compare” - (Geodigm Corporation, Chanhassen, MN) using automatic surface to surface registration [19].

**Fig. 2:**
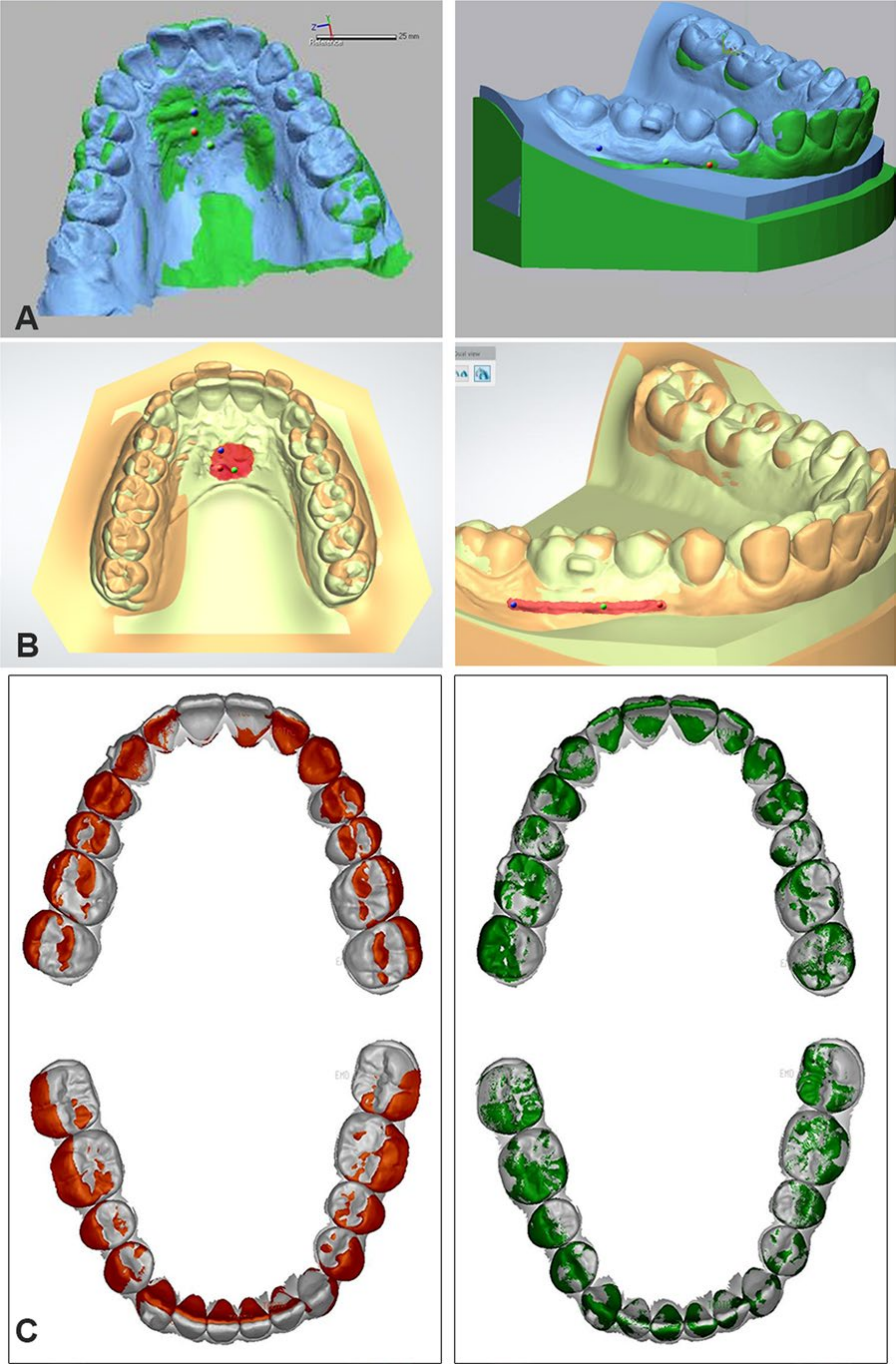
Registration of maxillary and mandibular models by (**A**) S-BF (**B**) I-SB (**C**) A-BF

The following steps were conducted before measurements were made:

1. Registration 2. Coordinate system generation 3. Measurement of tooth movement

1. Registration of the initial model and the digital setup using the three software packages Fig. 2

*Semiautomatic best fit registration software*: Landmark based registration was performed on stable rugae and mucogingival junction (MGJ) points, followed by global and fine regional best fit surface registration based on all points of the two models.

*Interactive surface-based registration software:* Registration was done using surface 3-point method which involved selection of the same landmarks on each of the corresponding models followed by painting an area of known stability to be used for surface-based registration.

*Automatic best fit registration:* Model trimming and segmentation of individual teeth of T2 was done. This was followed by global initial alignment based on three-points based on the mesial-buccal cusps of the first molars and the mesial-incisal point of the right central incisor. This initial registration was then refined by 30 iterations of a closest-point algorithm to achieve best fit of the occlusal surfaces. Finally, a best fit surface registration algorithm automatically superimposed individual teeth from the segmented T2 models on the corresponding teeth in the unsegmented T1 models.

2. Coordinate system generation

After registration, a three-dimensional (3D) coordinate system along the 3 principal axes were generated for tooth movement measurements. According to the software used, either model (S-BF and I-SB softwares) or tooth (A-BF) global reference frames were generated. Model global reference frames are defined as a coordinate system of three mutually perpendicular, intersecting axes (*x* = anteroposterior, *y* = occluso-gingival, and z = mediolateral). The “*x*-axis” is defined as the intersection of sagittal and occlusal planes, the “*y*-axis” as the intersection of the sagittal and coronal planes and the “*z*-axis” as the intersection of the coronal and occlusal planes [28]. The 3 D planes of space are the occlusal plane (XZ), midsagittal plane (XY), and the coronal plane (YZ).

For S-BF, one global model reference frame with the three mutually perpendicular intersecting axes (X, Y, Z) and orthogonal planes was constructed to measure all tooth movements (Composite Model Coordinates). On the other hand, for I-SB, each tooth required the generation of its own spatial model reference frame to individually measure tooth movements (Repeated Model Coordinates). However, for A-BF, a local tooth reference frame that the software automatically generates, defining the principal local coordinate tooth axes was generated (Automated Tooth Coordinates).

3. 3D tooth movement measurements

After all digital models (T1 & T2) were oriented in the same coordinate system via registration, it was possible to evaluate how the tooth positions changed. Registration of the T2 model onto the T1 model resulted in a 3 × 3 rotation matrix that described tooth movement. The change in the angular movement of each tooth between (T1) and (T2) was measured in degrees. The definitions used were as described by Daskalogiannakis et al. [28].

A. Tip: defined as rotation of a tooth around the labiolingual (x-axis) (when referring to an incisor), or around the buccolingual (z-axis) (when referring to a posterior tooth), thereby causing a change in its angulation.
B. Torque: defined as rotation of a tooth around its mesiodistal axis (z-axis) (when referring to an incisor), or around the (x-axis) (when referring to a posterior tooth), thereby causing a change in its inclination.
C. Rotation: defined as rotation of a tooth around its long axis; rotation in the x-z plane, around the y-axis.

The measured angular changes from DS were recorded in Excel (Microsoft Excel: 2016 Microsoft Corporation) for comparisons with similar measurements taken from the three studied software.

### Intra and inter-examiner reliability

Initially, one researcher (SA) performed all registrations of pretreatment scans with their digital setups, reference landmarks and axes identification, modification of local coordinates, as well as all tooth movement measurements. Another calibrated investigator (NV) repeated the measurements on 5 randomly selected scan sets for inter-operator reliability. Four weeks later the first researcher (SA) repeated measurements on 5 randomly selected scans to test intra-operator reliability. All measures were pooled to give a summary estimate to calculate Intra Class Correlation Coefficients for intra-examiner and inter-examiner reliability.

### Statistical analysis of the data

Statistical analysis was carried out using IBM SPSS software package version 20.0. (Armonk, NY: IBM Corp). Data from individual teeth were pooled to provide an overall estimate of the amount of tooth movement in each degree of freedom and summarized as mean and standard deviation. Two-way fixed-rater single-measure Intra Class Correlation Coefficient (ICC) of absolute agreement were calculated between the pooled amount of tooth movement in each degree of freedom measured by each software package and the amount of tooth movement from the digital setup (reference standard). Overall agreement between the three software packages were similarly calculated. Based on the 95% confidence  interval of the ICC estimate, values less than 0.5, between 0.5 and 0.75, between 0.75 and 0.9, and greater than 0.90 are indicative of poor, moderate, good, and excellent reliability, respectively [29]. Statistical significance of the obtained results was expressed at *p* ≤ 0.01 to account for multiple comparisons.

## Results

Excellent intra- and inter-examiner reliabilities were found for S-BF and A-BF software packages (intra-examiner reliability: 0.941, 0.978 respectively and inter-examiner reliability: 0.926, 0.944 respectively), while I-SB software showed good intra- and inter-examiner reliabilities for all the procedures (0.899, 0.798).

**Fig. 3:**
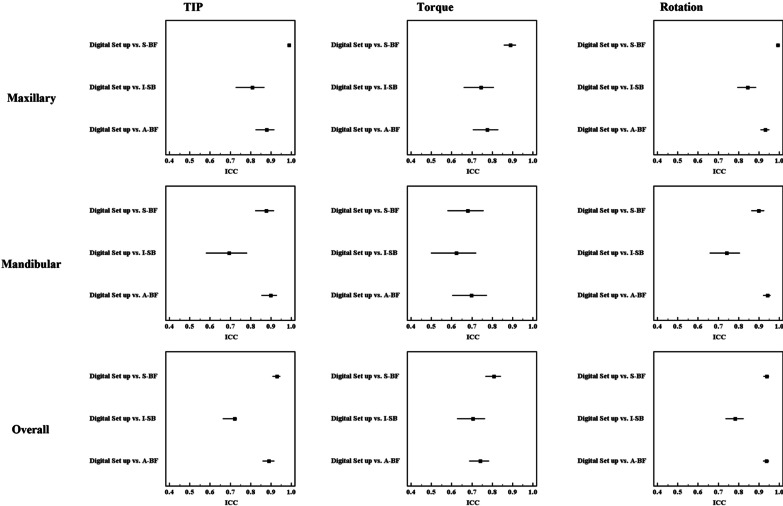
Forest plots of ICC of agreement between registration software packages and the digital setup for tip, torque and rotation

Table 2 shows the descriptive statistics (mean and standard deviation) for the maxillary and mandibular teeth with respect to the three angular movements for the DS and the three tested software packages. Agreement between each package and the reference standard are presented as ICC in Table 3 and as forest plots in Fig. 3.

**Table 2:** Amount of angular tooth movement determined by each software package

Type	Movements	No	Digital Setup Mean ± S.D	S-BF Mean ± S.D	I-SB Mean ± S.D	A-BF Mean ± S.D
Maxillary	Tip	97	− 1.043 ± 5.3	− 1.012 ± 4.9	− 0.435 ± 2	− 0.730 ± 3.6
	Torque	190	− 2.884 ± 4.5	− 2.572 ± 4.1	− 2.060 ± 3.7	− 2.258 ± 3.4
	Rotation	149	− 1.287 ± 9.8	− 1.235 ± 9.3	− 0.808 ± 7.3	− 1.219 ± 8.7
Mandibular	Tip	104	0.164 ± 5.6	0.060 ± 5	0.001 ± 2.7	0.067 ± 4.2
	Torque	143	− 1.906 ± 4.5	− 1.547 ± 3.6	− 0.986 ± 2.5	− 1.791 ± 4.2
	Rotation	146	0.302 ± 12.2	0.310 ± 11	0.525 ± 8.7	0.295 ± 12.1
Overall	Tip	201	− 0.418 ± 5.5	− 0.457 ± 5	− 0.209 ± 2.4	− 0.318 ± 3.9
	Torque	333	− 2.464 ± 4.5	− 2.132 ± 4	− 1.599 ± 3.3	− 2.057 ± 3.8
	Rotation	295	− 0.5007 ± 11.1	− 0.4703 ± 10	− 0.1483 ± 8	− 0.4696 ± 10.5

Data was expressed using Mean ± SD

**Table 3:** Intra Class Correlation Coefficient for different movements among the three software packages, in comparison to Digital Setup

Movements	Type	Digital setup versus S-BF		Digital setup versus I-SB		Digital setup versus A-BF	
		ICC	95% C. I	ICC	95% C. I	ICC	95% C. I
TIP	Maxillary	0.989*	0.983–0.992	0.808*	0.726–0.867	0.879*	0.825–0.917
	Mandibular	0.877*	0.823–0.915	0.694*	0.579–0.782	0.898*	0.853–0.930
	Overall	0.929*	0.908–0.946	0.720*	0.662–0.731	0.890*	0.858–0.916
Torque	Maxillary	0.890*	0.856–0.917	0.744*	0.660–0.808	0.775*	0.705–0.829
	Mandibular	0.679*	0.580–0.758	0.623*	0.498–0.720	0.697*	0.603–0.773
	Overall	0.808*	0.767–0.843	0.704*	0.626–0.765	0.740*	0.687–0.785
Rotation	Maxillary	0.993*	0.990–0.995	0.845*	0.793–0.886	0.932*	0.907–0.951
	Mandibular	0.899*	0.863–0.926	0.740*	0.657–0.806	0.942*	0.920–0.957
	Overall	0.938*	0.922–0.950	0.783*	0.735–0.824	0.936*	0.920–0.949

ICC, Intra Class Correlation Coefficient; CI, Confidence Interval; LL, Lower Limit; UL, Upper Limit

*All values were significant at *p* ≤ 0.001

For both S-BF and I-SB software, the mandibular ICCs values were lower than the maxillary ICCs across all measurements. This was not true for  A-BF software, which had mandibular ICC values greater than the maxillary equivalents for tip and rotation angles.

Rotation angle showed the highest agreement among all 3 tested software packages (Overall ICC S-BF: 0.938, I-SB: 0.783, A-BF: 0.936). Tip ICC values came second (Overall ICC S-BF: 0.929, I-SB: 0.720, A-BF: 0.890) and the least ICC values for the three tested software packages were for torque (Overall ICC S-BF: 0.808, I-SB: 0.704, A-BF: 0.740).

For S-BF software, the maxillary ICC for all angular measurements showed excellent agreement with the DS (> 0.90) except for maxillary torque ICC which showed only good agreement (0.890). The mandibular ICCs for all measurements showed good agreement (0.75–0.90) with the DS, except for the mandibular torque which showed only moderate agreement (0.679). In I-SB software, all maxillary angular measurements showed good agreement (0.75–0.90), except for the maxillary torque ICC value which showed moderate value of agreement (0.744). Additionally, all mandibular angular ICCs showed only moderate values for agreement (0.50–< 0.75). In A-BF software, the ICCs for the angular measurements differed, where the  maxillary and mandibular rotations showed excellent agreement (> 0.90), tip showed good agreement (0.75–0.90), and torque showed only moderate agreement (0.50–< 0.75).

Overall, S-BF software showed highest agreement, with reference to the DS, in all maxillary angular measurements, followed by A-BF software and then I-SB software respectively. A-BF software showed highest agreement for all mandibular angular measurements, followed by S-BF software and then I-SB software respectively.

## Discussion

Digital orthodontic solutions based on tooth movement simulations have become integral to planning, testing the efficacy of treatment techniques and quantifying treatment effects [30]. The measurement of the amount of orthodontic tooth movement is performed by registration software packages which may differ depending upon the registration algorithm used [10]. There is a paucity of studies testing the accuracy of different software packages used in the literature to compare treatment effects or determine technique efficacy [5, 8, 10, 19–21, 31–35].

Since tooth movements on the digital setup were performed by the principal investigator, the true value for translation and rotation *(type, direction and degree)* for each tooth could be used as a reference, as was reported by several previous studies in the literature [13, 20, 36]. The reliability, accuracy, and validity of using digital setup generated by OrthoAnalyzer software was previously evaluated in two studies and it was concluded that they are as effective and accurate as manual setups and represent an efficient tool for diagnosis that can be reliably reproduced [37, 38].

The present study used reference landmarks and area on the rugae for registration of the maxillary digital models in two of the three software (S-BF and I-SB software) that required reference structures. The selected landmarks have been documented previously in several studies to be considered as stable landmarks for maxillary digital model registration [11, 22, 23, 39]. As for the mandibular arch, posterior landmarks on the MGJ were used in the same software. This was based on the findings by Ioshida et al. [40] who reported good stability of MGJ to be used as a reference area. Contrastingly, A-BF software did not require the selection of a reference point or area for either arch outside the dentition.

Mandibular Intra Class Correlation Coefficient values (ICC) were steadily lower than their maxillary equivalents for all the movements in the two software (S-BF and I-SB) that required either landmark or surface selection for the registration. In contrast to A-BF software which showed higher mandibular ICCs for tip and rotation in comparison to their maxillary equivalents. Moreover, mandibular ICCs were always greater for A-BF software than the other two studied software. This can be explained by the fact that it does not require anatomical landmark or surface selection before registration but rather depends on the automatic superimposition tool of the software after initial global alignment. The software removes the interproximal papillae and model base apical to the gingival margin to ensure that the analysis is based solely on tooth-surface features. This implies that the mandibular superimposition using the MGJ landmarks with an area around it as a reference is less accurate than the maxillary superimposition using the rugae area. In existing literature, only two studies have attempted to study stable landmarks for accurate and reliable mandibular superimposition [24, 40]. Numerous studies however, have endorsed the accuracy of maxillary reference points and areas to be used for maxillary digital superimposition [11–13, 21, 22, 26, 39, 41–43].

Although the mucogingival line is a stable anatomic landmark that is not permanently altered by either orthodontics or surgery, the validated methods used in the study by Ioshida et al. [40] may have greater errors if teeth have been moved out of the alveolar bone (i.e., when an alveolar bone dehiscence is created) or if severe periodontal disease develops longitudinally. Moreover, limitations might become more evident if treatment includes a large amount of tooth movement (i.e., orthodontic expansion or a great amount of extrusion) and signs of gingival inflammation. Therefore, if one of these conditions are present, then a software like A-BF which doesn’t need gingival landmarks for registration, can be the preferred choice.

The current study employed registration techniques as mandated by the algorithms used in the software [16]. The superimposition approach for software S-BF and A-BF was a best-fit method [20, 22, 31, 42]. This technique of ‘fine matching’ uses thousands of reference points instead of a few landmarks/area and is based on ‘iterative closest point algorithms’ (ICP) [44]. The effect of outliers is reduced while accuracy markedly improves. Although I-SB software uses a surface-based method, it doesn’t use an algorithm that iterates to improve the quality of superimposition, unlike the ICP employed in S-BF and A-BF software. This explains their higher ICC values compared to I-SB software.

The lowest ICC values for all measured movements with I-SB software could be attributed to an important factor for digital superimpositions which is to have an accurate and reproducible coordinate system. S-BF software had one global coordinate system, A-BF software had automated computations for placement of local coordinate systems at each tooth’s approximate center of resistance [10, 19, 33], while I-SB software required creation of customized global coordinates for each tooth. One might assume that the method employed by this software will be more accurate because it is customized for each tooth according to its location in the dental arch. This, however, wasn’t true as it introduced more operator errors.

Rotation had the highest agreements amongst the angular measurements for the three software when compared to the setup. The current study evaluated the difference between T2 and T1 on external planes instead of using internal long axes for all angular measurements. Similar results to those reported in the present study have been documented by Chong et al. [35], who used an external reference plane. Another interpretation to the presented findings could be referred to the use of incisal edges and central grooves for projections, which are more reproducible compared to the long axes of teeth. Our findings contradict Grauer et al. [10], who found rotations to have the largest discrepancies, due to measurement of rotations along the long axis of a tooth. The tip angle was the second most accurate measurement amongst the three software. The tip was measured as a differential between T2 and T1 rather than absolute values, which explains minimal method errors. The measurement of torque angle, which is traditionally unreliable with study models, showed the lowest ICC values among all angular measurements in the three tested software. The location of precise tangents to labial surfaces has shown poor reproducibility conventionally. Ashmore et al. [41], found poor reliability for angular values. They ascribed measurement errors in digitization responsible for this finding. In their study, angular measurements relied on location of four molar points separately. In the present study, angular measurements were dependent on the location of long axes and not on individual landmarks.

Choosing the most efficient software to perform registrations is an important factor to consider when selecting between different software. Automatic best fit registration software was the most user-friendly software to use with the least time needed to complete the whole process, followed by the semiautomatic best fit registration software, with the interactive surface-based registration software coming third. This aspect, however, will be formally tested and reported in future publications. Agreements between the three software could also be evaluated in clear aligner therapy treatment by superimposing post treatment scans on simulations to test accuracy. Based on the conditions of the current study, the semiautomatic best fit registration software offers a greater advantage in terms of agreement to a reference standard compared to the others.

## Conclusions

1. Semiautomatic best fit registration software (S-BF) consistently showed excellent agreement in measuring the amount of tooth movement compared to the reference standard, whereas automatic best fit registration software (A-BF) and interactive surface-based registration software (I-SB) showed acceptable agreement. None of the studied software packages showed poor agreement.
2. Automatic best fit registration software (A-BF) showed higher values of agreement for mandibular measurements compared to the other software packages, whereas semiautomatic best fit registration software (S-BF) showed higher values of agreement for maxillary measurements.
3. Accuracy of digital model superimpositions for tooth movements studied in superimposition studies, can be attributed to the algorithm employed for quantification.

## Limitations of the present study

1. All measurements were based on the anatomy of the clinical crown due to the absence of roots in intraoral scans, hence the tooth centroid could not be defined. The angular measurements represent rotation of the long axis of the clinical crown in the 3 planes of space, thus it will not account for situations where there is a discordance between the long axis of the clinical crown and the root.
2. Using the digital setup as a reference standard maximize the chance of agreement with the registration software since the adjacent soft tissues are not altered. Accounting for the tissue changes concomitant with orthodontic tooth movement, the accuracy of the registration software packages could be lower.

## Data Availability

The datasets used and/or analyzed during the current study are available from the corresponding author on reasonable request.
